# (±)-*trans*-5-Benzoyl-2-(1*H*-indol-3-yl)-4-phenyl-4,5-dihydro­furan-3-carbonitrile

**DOI:** 10.1107/S1600536812018430

**Published:** 2012-04-28

**Authors:** J. Suresh, R. Vishnupriya, P. Gunasekaran, S. Perumal, P. L. Nilantha Lakshman

**Affiliations:** a Department of Physics, The Madura College, Madurai 625 011, India; b Department of Organic Chemistry, School of Chemistry, Madurai Kamaraj University, Madurai 625 021, India; c Department of Food Science and Technology, University of Ruhuna, Mapalana, Kamburupitiya 81100, Sri Lanka

## Abstract

The furan ring in the title compound, C_26_H_18_N_2_O_2_, is twisted about the C(H)—C(H) bond. The mol­ecular structure is stabilized by an intra­molecular C—H⋯O inter­action, which generates an *S*(6) ring motif. The presence of N—H⋯N hydrogen bonds leads to inversion dimers, which are stabilized in the crystal packing by C—H⋯O and C—H⋯π inter­actions, forming layers that stack along the *a* axis.

## Related literature

For graph-set notation, see: Bernstein *et al.* (1995 ▶). For the importance of furan derivatives, see: Kappe *et al.* (1997 ▶); Sato *et al.* (1999 ▶); Smith *et al.* (2002 ▶). For additional conformation analysis, see: Cremer & Pople (1975 ▶).
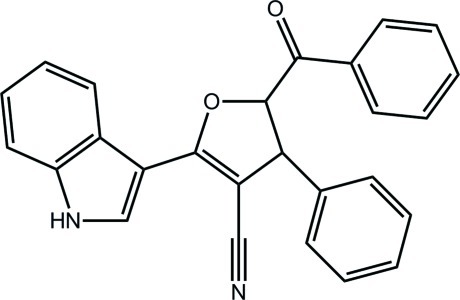


## Experimental

### 

#### Crystal data

C_26_H_18_N_2_O_2_
*M* *_r_* = 390.42
Monoclinic, 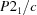

*a* = 12.4027 (5) Å
*b* = 8.3722 (4) Å
*c* = 19.7472 (8) Å
β = 107.570 (2)°
*V* = 1954.85 (15) Å^3^
*Z* = 4
Mo *K*α radiation
μ = 0.09 mm^−1^
*T* = 293 K
0.17 × 0.14 × 0.13 mm

#### Data collection

Bruker Kappa APEXII diffractometer
Absorption correction: multi-scan (*SADABS*; Sheldrick, 1996 ▶) *T*_min_ = 0.967, *T*_max_ = 0.974
20260 measured reflections
4403 independent reflections
3021 reflections with *I* > 2σ(*I*)
*R*_int_ = 0.030

#### Refinement

*R*[*F* ^2^ > 2σ(*F* ^2^)] = 0.042
*wR*(*F* ^2^) = 0.117
*S* = 1.02
4403 reflections
271 parameters
H-atom parameters constrained
Δρ_max_ = 0.16 e Å^−3^
Δρ_min_ = −0.15 e Å^−3^

### 

Data collection: *APEX2* (Bruker, 2004 ▶); cell refinement: *SAINT* (Bruker, 2004 ▶); data reduction: *SAINT*; program(s) used to solve structure: *SHELXS97* (Sheldrick, 2008 ▶); program(s) used to refine structure: *SHELXL97* (Sheldrick, 2008 ▶); molecular graphics: *PLATON* (Spek, 2009 ▶); software used to prepare material for publication: *SHELXL97*.

## Supplementary Material

Crystal structure: contains datablock(s) global, I. DOI: 10.1107/S1600536812018430/tk5086sup1.cif

Structure factors: contains datablock(s) I. DOI: 10.1107/S1600536812018430/tk5086Isup2.hkl

Supplementary material file. DOI: 10.1107/S1600536812018430/tk5086Isup3.cml

Additional supplementary materials:  crystallographic information; 3D view; checkCIF report

## Figures and Tables

**Table 1:** Hydrogen-bond geometry (Å, °) *Cg*1 is the centroid of the N1,C31,C32,C37,C38 ring.

*D*—H⋯*A*	*D*—H	H⋯*A*	*D*⋯*A*	*D*—H⋯*A*
C33—H33⋯O1	0.93	2.56	3.040 (2)	112
C56—H56⋯O2^i^	0.93	2.45	3.330 (2)	158
N1—H1⋯N2^ii^	0.86	2.20	3.037 (2)	163
C43—H43⋯*Cg*1^iii^	0.93	2.96	3.410 (2)	112

Symmetry codes: (i) 
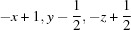
; (ii) 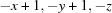; (iii) 
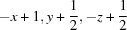
.

**Fig. 1.**
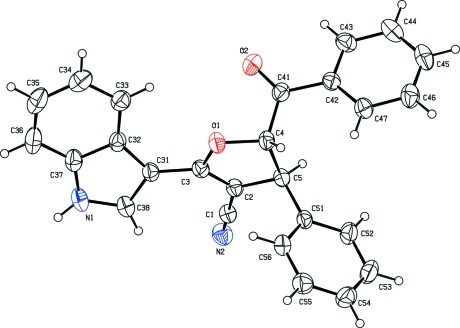
The molecular structure of (I), showing 30% probability displacement ellipsoids and the atom-numbering scheme.

**Fig. 2.**
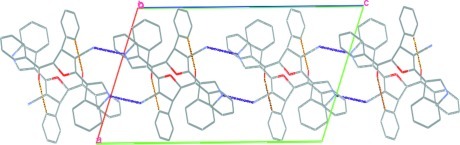
The packing diagram of the molecule (I). The C—H···O interactions are shown as dashed lines.

## supplementary crystallographic information

### Comment

Benzofurans have physiological, pharmacological and toxic properties, and there is continuing interest in their synthesis (Kappe *et al.*, 1997). Various benzofuran derivatives have been investigated as estrogen receptor ligands, because selective estrogen receptor modulators such as ralixofene have emerged as potential therapeutics for the prevention and treatment of osteoporosis (Sato *et al.*, 1999; Smith *et al.*, 2002). In view of their high medicinal value, and in conjunction with our research interests, we were prompted to synthesize and report the X-ray structure determination of the title compound, (I).

In the title compound (Fig. 1), the five-membered furanyl ring adopts a twisted conformation as evident from the puckering parameters (Cremer & Pople, 1975): Q = 0.1429 (2) Å and φ = 126.0 (6)°. The five-(N2,/C38/C31/C32/C37) and six-membered (C32—C37) rings in the indole group are planar, with a dihedral angle of 0.95 (1)° between them. The dihedral angle between the phenyl rings (C42—C47 and C51—C56) is 31.56 (1)°. The molecular structure is stabilized by an intramolecular C—H···O interaction which generates an *S*(6) ring motif (Bernstein *et al.*, 1995).

The presence of N—H···N hydrogen bonds leads to inversion dimers which are stabilised in the crystal packing by C—H···O and C—H···π interactions, Table 1, to form layers that stack along the *a* axis, Fig. 2.

### Experimental

To a stirred mixture of 2-(1*H*-indole-3-carbonyl)-3-phenylacrylonitrile (1.0 eq.) and phenacylpyridinium bromide (1.0 eq.) in water (10 ml) was added drop-wise triethylamine (0.25 eq.) at room temperature. The resulting clear solution, that slowly became turbid, was stirred at room temperature for 0.5 h. Then the separated free-flowing solid was filtered and washed with methanol (3 ml) to afford the title compound as pale-yellow solids. The product was recrystallized from EtOH/EtOAc mixture (1:1 ratio *v*/*v* ml) to give pure compound, as pale-yellow crystals. *M*. pt: 521 K; Yield: 88%.

### Refinement

H atoms were placed at calculated positions and allowed to ride on their carrier atoms with N—H = 0.86 Å and C—H = 0.93–0.98 Å, and with *U*_iso_ = 1.2*U*_eq_(N,C) for CH and *U*_iso_ = 1.5*U*_eq_(C) for CH_3_.

### Crystal data

**Table:**

C_26_H_18_N_2_O_2_	*F*(000) = 816
*M**_r_* = 390.42	*D*_x_ = 1.327 Mg m^−^^3^
Monoclinic, *P*2_1_/*c*	Mo *K*α radiation, λ = 0.71073 Å
Hall symbol: -P 2ybc	Cell parameters from 2000 reflections
*a* = 12.4027 (5) Å	θ = 2–31°
*b* = 8.3722 (4) Å	µ = 0.09 mm^−^^1^
*c* = 19.7472 (8) Å	*T* = 293 K
β = 107.570 (2)°	Block, pale-yellow
*V* = 1954.85 (15) Å^3^	0.17 × 0.14 × 0.13 mm
*Z* = 4	

### Data collection

**Table:**

Bruker Kappa APEXII diffractometer	4403 independent reflections
Radiation source: fine-focus sealed tube	3021 reflections with *I* > 2σ(*I*)
Graphite monochromator	*R*_int_ = 0.030
Detector resolution: 0 pixels mm^-1^	θ_max_ = 27.3°, θ_min_ = 2.2°
ω and φ scans	*h* = −12→16
Absorption correction: multi-scan (*SADABS*; Sheldrick, 1996)	*k* = −10→10
*T*_min_ = 0.967, *T*_max_ = 0.974	*l* = −25→25
20260 measured reflections	

### Refinement

**Table:**

Refinement on *F*^2^	Primary atom site location: structure-invariant direct methods
Least-squares matrix: full	Secondary atom site location: difference Fourier map
*R*[*F*^2^ > 2σ(*F*^2^)] = 0.042	Hydrogen site location: inferred from neighbouring sites
*wR*(*F*^2^) = 0.117	H-atom parameters constrained
*S* = 1.02	*w* = 1/[σ^2^(*F*_o_^2^) + (0.0537*P*)^2^ + 0.2502*P*] where *P* = (*F*_o_^2^ + 2*F*_c_^2^)/3
4403 reflections	(Δ/σ)_max_ = 0.001
271 parameters	Δρ_max_ = 0.16 e Å^−^^3^
0 restraints	Δρ_min_ = −0.15 e Å^−^^3^

### Special details

**Table:**

Geometry. All e.s.d.'s (except the e.s.d. in the dihedral angle between two l.s. planes) are estimated using the full covariance matrix. The cell e.s.d.'s are taken into account individually in the estimation of e.s.d.'s in distances, angles and torsion angles; correlations between e.s.d.'s in cell parameters are only used when they are defined by crystal symmetry. An approximate (isotropic) treatment of cell e.s.d.'s is used for estimating e.s.d.'s involving l.s. planes.
Refinement. Refinement of *F*^2^ against ALL reflections. The weighted *R*-factor *wR* and goodness of fit *S* are based on *F*^2^, conventional *R*-factors *R* are based on *F*, with *F* set to zero for negative *F*^2^. The threshold expression of *F*^2^ > σ(*F*^2^) is used only for calculating *R*-factors(gt) *etc*. and is not relevant to the choice of reflections for refinement. *R*-factors based on *F*^2^ are statistically about twice as large as those based on *F*, and *R*- factors based on ALL data will be even larger.

### Fractional atomic coordinates and isotropic or equivalent isotropic displacement parameters (Å^2^)

**Table:**

	*x*	*y*	*z*	*U*_iso_*/*U*_eq_	
C1	0.67286 (12)	0.45451 (19)	0.17299 (7)	0.0470 (3)	
C2	0.63051 (11)	0.34109 (17)	0.21081 (7)	0.0444 (3)	
C3	0.52658 (12)	0.27563 (17)	0.18813 (7)	0.0450 (3)	
C4	0.59768 (11)	0.18757 (18)	0.30149 (7)	0.0460 (3)	
H4	0.6215	0.0796	0.3187	0.055*	
C5	0.69611 (11)	0.27397 (18)	0.28285 (7)	0.0446 (3)	
H5	0.7251	0.3614	0.3164	0.054*	
C31	0.43701 (12)	0.28633 (17)	0.12162 (7)	0.0463 (3)	
C32	0.32001 (12)	0.24303 (17)	0.10900 (8)	0.0484 (4)	
C33	0.25537 (13)	0.1932 (2)	0.15212 (9)	0.0598 (4)	
H33	0.2878	0.1823	0.2009	0.072*	
C34	0.14258 (15)	0.1608 (2)	0.12050 (11)	0.0744 (5)	
H34	0.0985	0.1280	0.1486	0.089*	
C35	0.09299 (16)	0.1758 (3)	0.04764 (12)	0.0834 (6)	
H35	0.0169	0.1508	0.0279	0.100*	
C36	0.15389 (16)	0.2265 (2)	0.00444 (10)	0.0766 (6)	
H36	0.1205	0.2373	−0.0443	0.092*	
C37	0.26741 (13)	0.26136 (19)	0.03599 (8)	0.0569 (4)	
C38	0.44868 (14)	0.32868 (19)	0.05720 (8)	0.0549 (4)	
H38	0.5155	0.3629	0.0496	0.066*	
C41	0.56106 (12)	0.27837 (19)	0.35731 (8)	0.0491 (4)	
C42	0.64154 (12)	0.28240 (18)	0.43077 (7)	0.0464 (3)	
C43	0.62343 (14)	0.3942 (2)	0.47757 (8)	0.0593 (4)	
H43	0.5625	0.4641	0.4627	0.071*	
C44	0.69484 (17)	0.4028 (2)	0.54600 (9)	0.0731 (5)	
H44	0.6822	0.4786	0.5772	0.088*	
C45	0.78458 (17)	0.3001 (2)	0.56826 (9)	0.0728 (5)	
H45	0.8327	0.3062	0.6146	0.087*	
C46	0.80372 (15)	0.1881 (2)	0.52240 (9)	0.0679 (5)	
H46	0.8645	0.1182	0.5377	0.081*	
C47	0.73263 (13)	0.1793 (2)	0.45343 (8)	0.0567 (4)	
H47	0.7460	0.1040	0.4223	0.068*	
C51	0.79172 (11)	0.16457 (18)	0.28071 (7)	0.0448 (3)	
C52	0.90157 (12)	0.1926 (2)	0.32275 (8)	0.0612 (4)	
H52	0.9172	0.2801	0.3532	0.073*	
C53	0.98818 (14)	0.0916 (3)	0.31985 (10)	0.0726 (5)	
H53	1.0616	0.1117	0.3484	0.087*	
C54	0.96707 (15)	−0.0370 (2)	0.27562 (9)	0.0685 (5)	
H54	1.0259	−0.1034	0.2732	0.082*	
C55	0.85815 (15)	−0.0680 (2)	0.23456 (9)	0.0661 (5)	
H55	0.8430	−0.1567	0.2049	0.079*	
C56	0.77161 (13)	0.0315 (2)	0.23725 (8)	0.0556 (4)	
H56	0.6982	0.0090	0.2094	0.067*	
N1	0.34776 (12)	0.31303 (17)	0.00616 (6)	0.0629 (4)	
H1	0.3359	0.3326	−0.0382	0.076*	
N2	0.70692 (12)	0.54844 (18)	0.14254 (7)	0.0630 (4)	
O1	0.50438 (8)	0.17832 (12)	0.23712 (5)	0.0523 (3)	
O2	0.47216 (10)	0.34802 (17)	0.34197 (6)	0.0765 (4)	

### Atomic displacement parameters (Å^2^)

**Table:**

	*U*^11^	*U*^22^	*U*^33^	*U*^12^	*U*^13^	*U*^23^
C1	0.0484 (8)	0.0492 (9)	0.0412 (7)	0.0003 (6)	0.0104 (6)	0.0016 (7)
C2	0.0464 (7)	0.0463 (8)	0.0394 (7)	0.0010 (6)	0.0112 (6)	0.0031 (6)
C3	0.0495 (8)	0.0443 (8)	0.0401 (7)	0.0024 (6)	0.0120 (6)	0.0019 (6)
C4	0.0439 (7)	0.0510 (9)	0.0386 (7)	−0.0013 (6)	0.0056 (6)	0.0071 (6)
C5	0.0459 (7)	0.0485 (8)	0.0365 (7)	−0.0046 (6)	0.0081 (6)	0.0004 (6)
C31	0.0492 (8)	0.0453 (8)	0.0407 (7)	0.0048 (6)	0.0077 (6)	−0.0002 (6)
C32	0.0478 (8)	0.0429 (8)	0.0480 (8)	0.0054 (6)	0.0048 (6)	−0.0030 (6)
C33	0.0540 (9)	0.0594 (11)	0.0638 (10)	0.0033 (7)	0.0146 (8)	−0.0020 (8)
C34	0.0556 (10)	0.0716 (12)	0.0970 (14)	−0.0004 (9)	0.0246 (10)	−0.0079 (11)
C35	0.0495 (10)	0.0815 (14)	0.1046 (16)	0.0016 (9)	0.0012 (11)	−0.0131 (12)
C36	0.0620 (11)	0.0755 (13)	0.0698 (12)	0.0075 (9)	−0.0140 (9)	−0.0043 (10)
C37	0.0569 (9)	0.0504 (9)	0.0531 (9)	0.0066 (7)	0.0009 (7)	−0.0010 (7)
C38	0.0605 (9)	0.0560 (10)	0.0432 (8)	−0.0007 (7)	0.0081 (7)	0.0017 (7)
C41	0.0444 (8)	0.0544 (9)	0.0477 (8)	0.0006 (7)	0.0128 (6)	0.0101 (7)
C42	0.0485 (8)	0.0507 (9)	0.0414 (7)	−0.0031 (6)	0.0157 (6)	0.0091 (6)
C43	0.0688 (10)	0.0625 (10)	0.0503 (9)	0.0030 (8)	0.0235 (8)	0.0045 (8)
C44	0.0986 (14)	0.0742 (13)	0.0470 (9)	−0.0058 (11)	0.0226 (9)	−0.0028 (9)
C45	0.0854 (13)	0.0796 (13)	0.0441 (9)	−0.0160 (10)	0.0055 (8)	0.0071 (9)
C46	0.0655 (10)	0.0733 (12)	0.0560 (10)	0.0025 (9)	0.0050 (8)	0.0166 (9)
C47	0.0604 (9)	0.0595 (10)	0.0472 (8)	0.0034 (7)	0.0118 (7)	0.0077 (7)
C51	0.0427 (7)	0.0535 (9)	0.0351 (7)	−0.0030 (6)	0.0071 (5)	0.0062 (6)
C52	0.0477 (8)	0.0690 (11)	0.0578 (9)	−0.0054 (8)	0.0020 (7)	−0.0060 (8)
C53	0.0426 (8)	0.0892 (14)	0.0756 (11)	0.0024 (9)	0.0022 (8)	0.0033 (11)
C54	0.0583 (10)	0.0767 (13)	0.0704 (11)	0.0155 (9)	0.0194 (8)	0.0117 (10)
C55	0.0697 (11)	0.0649 (11)	0.0615 (10)	0.0089 (9)	0.0167 (8)	−0.0048 (8)
C56	0.0491 (8)	0.0642 (10)	0.0470 (8)	−0.0022 (7)	0.0049 (6)	−0.0029 (8)
N1	0.0715 (9)	0.0669 (9)	0.0396 (7)	0.0017 (7)	0.0006 (6)	0.0059 (6)
N2	0.0694 (9)	0.0656 (9)	0.0540 (8)	−0.0076 (7)	0.0185 (7)	0.0088 (7)
O1	0.0495 (6)	0.0612 (7)	0.0406 (5)	−0.0114 (5)	0.0055 (4)	0.0070 (5)
O2	0.0551 (7)	0.1000 (10)	0.0680 (7)	0.0243 (7)	0.0089 (6)	−0.0007 (7)

### Geometric parameters (Å, º)

**Table:**

C1—N2	1.1454 (18)	C38—H38	0.9300
C1—C2	1.404 (2)	C41—O2	1.2024 (17)
C2—C3	1.3469 (19)	C41—C42	1.4925 (19)
C2—C5	1.5174 (18)	C42—C43	1.380 (2)
C3—O1	1.3552 (17)	C42—C47	1.385 (2)
C3—C31	1.4443 (19)	C43—C44	1.376 (2)
C4—O1	1.4398 (15)	C43—H43	0.9300
C4—C41	1.517 (2)	C44—C45	1.370 (3)
C4—C5	1.5558 (19)	C44—H44	0.9300
C4—H4	0.9800	C45—C46	1.373 (3)
C5—C51	1.509 (2)	C45—H45	0.9300
C5—H5	0.9800	C46—C47	1.383 (2)
C31—C38	1.370 (2)	C46—H46	0.9300
C31—C32	1.442 (2)	C47—H47	0.9300
C32—C33	1.398 (2)	C51—C56	1.382 (2)
C32—C37	1.400 (2)	C51—C52	1.3847 (19)
C33—C34	1.376 (2)	C52—C53	1.381 (2)
C33—H33	0.9300	C52—H52	0.9300
C34—C35	1.389 (3)	C53—C54	1.361 (3)
C34—H34	0.9300	C53—H53	0.9300
C35—C36	1.367 (3)	C54—C55	1.374 (2)
C35—H35	0.9300	C54—H54	0.9300
C36—C37	1.388 (2)	C55—C56	1.372 (2)
C36—H36	0.9300	C55—H55	0.9300
C37—N1	1.372 (2)	C56—H56	0.9300
C38—N1	1.3557 (19)	N1—H1	0.8600
			
N2—C1—C2	179.19 (17)	O2—C41—C42	121.82 (14)
C3—C2—C1	124.91 (13)	O2—C41—C4	120.85 (13)
C3—C2—C5	110.46 (12)	C42—C41—C4	117.29 (12)
C1—C2—C5	124.63 (12)	C43—C42—C47	119.23 (14)
C2—C3—O1	112.80 (12)	C43—C42—C41	118.02 (13)
C2—C3—C31	132.33 (14)	C47—C42—C41	122.75 (14)
O1—C3—C31	114.84 (12)	C44—C43—C42	120.44 (16)
O1—C4—C41	109.35 (11)	C44—C43—H43	119.8
O1—C4—C5	107.16 (10)	C42—C43—H43	119.8
C41—C4—C5	111.55 (12)	C45—C44—C43	120.12 (17)
O1—C4—H4	109.6	C45—C44—H44	119.9
C41—C4—H4	109.6	C43—C44—H44	119.9
C5—C4—H4	109.6	C44—C45—C46	120.17 (16)
C51—C5—C2	113.71 (11)	C44—C45—H45	119.9
C51—C5—C4	113.81 (12)	C46—C45—H45	119.9
C2—C5—C4	99.04 (10)	C45—C46—C47	120.03 (17)
C51—C5—H5	109.9	C45—C46—H46	120.0
C2—C5—H5	109.9	C47—C46—H46	120.0
C4—C5—H5	109.9	C46—C47—C42	120.01 (16)
C38—C31—C32	106.70 (12)	C46—C47—H47	120.0
C38—C31—C3	126.34 (14)	C42—C47—H47	120.0
C32—C31—C3	126.81 (13)	C56—C51—C52	117.97 (14)
C33—C32—C37	119.04 (14)	C56—C51—C5	120.70 (12)
C33—C32—C31	134.57 (13)	C52—C51—C5	121.32 (14)
C37—C32—C31	106.39 (14)	C53—C52—C51	120.55 (16)
C34—C33—C32	118.29 (16)	C53—C52—H52	119.7
C34—C33—H33	120.9	C51—C52—H52	119.7
C32—C33—H33	120.9	C54—C53—C52	120.60 (15)
C33—C34—C35	121.58 (19)	C54—C53—H53	119.7
C33—C34—H34	119.2	C52—C53—H53	119.7
C35—C34—H34	119.2	C53—C54—C55	119.50 (16)
C36—C35—C34	121.28 (17)	C53—C54—H54	120.3
C36—C35—H35	119.4	C55—C54—H54	120.3
C34—C35—H35	119.4	C56—C55—C54	120.24 (17)
C35—C36—C37	117.53 (17)	C56—C55—H55	119.9
C35—C36—H36	121.2	C54—C55—H55	119.9
C37—C36—H36	121.2	C55—C56—C51	121.11 (14)
N1—C37—C36	130.07 (16)	C55—C56—H56	119.4
N1—C37—C32	107.68 (13)	C51—C56—H56	119.4
C36—C37—C32	122.24 (17)	C38—N1—C37	109.80 (13)
N1—C38—C31	109.43 (15)	C38—N1—H1	125.1
N1—C38—H38	125.3	C37—N1—H1	125.1
C31—C38—H38	125.3	C3—O1—C4	108.40 (10)
			
N2—C1—C2—C3	91 (13)	O1—C4—C41—O2	−10.0 (2)
N2—C1—C2—C5	−90 (13)	C5—C4—C41—O2	108.38 (16)
C1—C2—C3—O1	−175.50 (13)	O1—C4—C41—C42	172.28 (11)
C5—C2—C3—O1	5.01 (17)	C5—C4—C41—C42	−69.37 (16)
C1—C2—C3—C31	6.4 (3)	O2—C41—C42—C43	−13.3 (2)
C5—C2—C3—C31	−173.05 (15)	C4—C41—C42—C43	164.41 (13)
C3—C2—C5—C51	109.37 (14)	O2—C41—C42—C47	166.66 (16)
C1—C2—C5—C51	−70.13 (18)	C4—C41—C42—C47	−15.6 (2)
C3—C2—C5—C4	−11.72 (15)	C47—C42—C43—C44	−0.2 (2)
C1—C2—C5—C4	168.78 (14)	C41—C42—C43—C44	179.82 (15)
O1—C4—C5—C51	−106.79 (12)	C42—C43—C44—C45	−0.1 (3)
C41—C4—C5—C51	133.55 (12)	C43—C44—C45—C46	0.1 (3)
O1—C4—C5—C2	14.22 (14)	C44—C45—C46—C47	0.3 (3)
C41—C4—C5—C2	−105.43 (12)	C45—C46—C47—C42	−0.6 (3)
C2—C3—C31—C38	21.4 (3)	C43—C42—C47—C46	0.5 (2)
O1—C3—C31—C38	−156.61 (14)	C41—C42—C47—C46	−179.48 (14)
C2—C3—C31—C32	−163.71 (16)	C2—C5—C51—C56	−56.54 (18)
O1—C3—C31—C32	18.3 (2)	C4—C5—C51—C56	55.88 (17)
C38—C31—C32—C33	−178.57 (17)	C2—C5—C51—C52	124.44 (15)
C3—C31—C32—C33	5.7 (3)	C4—C5—C51—C52	−123.14 (15)
C38—C31—C32—C37	0.40 (16)	C56—C51—C52—C53	1.3 (2)
C3—C31—C32—C37	−175.29 (14)	C5—C51—C52—C53	−179.67 (15)
C37—C32—C33—C34	1.4 (2)	C51—C52—C53—C54	0.0 (3)
C31—C32—C33—C34	−179.72 (16)	C52—C53—C54—C55	−1.2 (3)
C32—C33—C34—C35	0.3 (3)	C53—C54—C55—C56	1.1 (3)
C33—C34—C35—C36	−1.3 (3)	C54—C55—C56—C51	0.3 (3)
C34—C35—C36—C37	0.5 (3)	C52—C51—C56—C55	−1.4 (2)
C35—C36—C37—N1	179.66 (18)	C5—C51—C56—C55	179.51 (14)
C35—C36—C37—C32	1.3 (3)	C31—C38—N1—C37	0.49 (18)
C33—C32—C37—N1	179.05 (14)	C36—C37—N1—C38	−178.77 (17)
C31—C32—C37—N1	−0.11 (17)	C32—C37—N1—C38	−0.22 (18)
C33—C32—C37—C36	−2.3 (2)	C2—C3—O1—C4	5.08 (16)
C31—C32—C37—C36	178.58 (15)	C31—C3—O1—C4	−176.50 (12)
C32—C31—C38—N1	−0.55 (17)	C41—C4—O1—C3	108.41 (13)
C3—C31—C38—N1	175.17 (14)	C5—C4—O1—C3	−12.65 (15)

### Hydrogen-bond geometry (Å, º)

Cg1 is the centroid of the N1,C31,C32,C37,C38 ring.

**Table:**

*D*—H···*A*	*D*—H	H···*A*	*D*···*A*	*D*—H···*A*
C33—H33···O1	0.93	2.56	3.040 (2)	112
C56—H56···O2^i^	0.93	2.45	3.330 (2)	158
N1—H1···N2^ii^	0.86	2.20	3.037 (2)	163
C43—H43···*Cg*1^iii^	0.93	2.96	3.410 (2)	112

Symmetry codes: (i) −*x*+1, *y*−1/2, −*z*+1/2; (ii) −*x*+1, −*y*+1, −*z*; (iii) −*x*+1, *y*+1/2, −*z*+1/2.
